# Confidence in the neurological examination and exam avoidance among medical residents

**DOI:** 10.1371/journal.pone.0360249

**Published:** 2026-10-08

**Authors:** Alexa B. Keeling, Vijay J. Daniels

**Affiliations:** 1 Division of Neurology, Department of Medicine, University of Alberta, Edmonton, Alberta, Canada; 2 Division of General Internal Medicine, Department of Medicine, University of Alberta, Edmonton, Alberta, Canada; AIIMS: All India Institute of Medical Sciences, INDIA

## Abstract

**Background and objectives:**

Neurophobia is the fear of clinical neurology. Little research has explored how the neurological exam contributes to this fear. We wanted to better understand the confidence that medical residents have with the neurological exam and determine how confidence affects their examination of neurological patients.

**Methods:**

This was a single-centre study in which Internal Medicine and Family Medicine residents rated their confidence in conducting different aspects of a neurological evaluation through a self-administered survey. Low confidence was defined as a score of ≤3 on a 5-point Likert scale. Participants also rated the likelihood of their peers skipping a relevant exam for patients presenting with neurological, cardiac, respiratory, gastrointestinal, or musculoskeletal complaints. Free form responses were sought to understand reasons for skipping.

**Results:**

There were 97 respondents (35 IM; 61 FM), out of a total of 350 eligible participants. 36 were first year, 45 were second year, and 14 were third year residents. Participants were least confident in localizing (Median = 3; IQR 2–4) and forming a differential (Median 4; IQR 3–4). Confidence did not improve with additional years of training and respondents felt increased exposure to neurology and more teaching would improve their confidence. Participants felt their peers were most likely to skip a neurological (Median = 3; IQR 2–3) or musculoskeletal exam (Median 3; IQR 2–4). Likelihood of skipping part of a neurological exam increased from post-graduate year one to two. Low confidence and lack of time were cited as major contributors.

**Conclusion:**

Residents appear to be less confident in conducting the non-physical aspects of the neurological exam, including localizing and forming a differential. Low confidence was a major contributor to residents forgoing a neurological exam. More teaching and exposure to neurology were the most common self-reported factors that would improve resident confidence in the physical exam.

## Introduction

The idea of “neurophobia” was first proposed by R. Jozefowicz in a 1994 paper where he used his anecdotal experiences to describe medical student’s fear of applying basic neuroscience to clinical neurology [1]. Since this seminal paper, we have learned neurophobia is not strictly limited to medical students – this fear of neurology is more pervasive and has been seen at the intern and resident level [2–4], even up to the level of the staff physician [5,6].

Neuroanatomy and the teaching of basic neuroscience seem to be the most prominent drivers of the fear of neurology [3,7] and thus seem to be the central focus in much of the current neurophobia research. Though identified to a lesser extent, the complex physical exam is still cited by many to be a contributing factor to neurophobia [2,8,9] – which likely explains why few studies have evaluated this topic further [10]. In a recent survey conducted on Internal Medicine residents, only 50% of residents were somewhat to very confident in conducting and interpreting the neurological exam [11]. However, this study did not further assess the reasons behind low confidence on the examination, or what parts of the examination were major contributors to lower confidence. When we tease apart the neurological evaluation, it becomes more than just the physical process of examining a patient [12]. One must be able to determine from the history what they should be evaluating for, identify key pathology from the examination, localize the issue from the findings, and finally generate a differential [12].

From prior research, we know that medical learners seem to have a good understanding of the essential components of a neurological exam. A study conducted in Montreal sought to understand if uncertainty in knowing the different aspects of the neurological exam was leading to anxiety in conducting the exam [10]. Moore and Chalk surveyed graduating medical students on what elements of the neurological exam should commonly be completed when evaluating for a neurologic concern and compared that to the same survey of Canadian neurologists. They found there was a high degree of consensus between the medical students and the licensed neurologists, indicating the students had an appropriate understanding of the exam components. In conclusion, the authors of this study felt that the anxiety that medical students feel in relation to the neurologic exam must arise from another source such as the actual mechanics of completing an exam or interpretation of the findings. Further studies have not yet evaluated confidence in the physical mechanics of completing the exam, the interpretation of exam findings, or the localization and differential that follow. Hence, it continues to remain unclear what parts of the examination process reduce learner confidence and lead to anxiety. Our study was therefore aimed at understanding what components of the neurological examination, both physical and non-physical, were contributing to lower confidence in conducting an examination

Equally as important, we wanted to understand how we could improve confidence levels in conducting a physical examination in neurological patients. When medical learners were surveyed on what would improve their confidence in neurology in general, learners wanted more exposure to neurological patients [13–15]. Interestingly, residents reported higher confidence and found neurology less difficult when compared to medical students [13]. One possible explanation for this could be increased exposure to neurological patients clinically and therefore more neurological pathology. In this study, we hypothesized that a major contributor to lack of confidence stems from difficulty identifying abnormal findings from a neurological examination.

An aspect of neurophobia research that has yet to be explored is understanding if a lack of confidence in one’s exam skills affects their willingness to complete a neurological exam – i.e., is neurophobia causing people to forgo examining neurological patients. This has been suggested as a possible side effect of neurophobia [11] but no study has yet assessed the validity of this claim. What we do know from past research is that medical students and residents have lower confidence managing neurological complaints [4,13,15], including emergent complaints [2], when compared to patients with complaints involving other organ systems. We also know that delaying patient assessment could contribute to a significant increase to morbidity and mortality, especially when we look at outcomes related to in-hospital stroke [16,17]. As such, in this study, we wanted to understand if lower confidence was a reason for medical learners to skip an examination of a neurological patient.

Taken together, the purpose of the study was to explore 1) learner’s confidence with different aspects of conducting a neurological examination, 2) how willingness to do a neurological examination related to confidence, and 3) associations with these factors and year of residency and primary discipline (Family Medicine (FM) and Internal Medicine (IM)).

## Methods

### Participants & consent

This was a single center study carried out at the University of Alberta in Edmonton, Canada. All residents, between June 23 and July 16, 2024, in the IM program (post-graduate year (PGY) 1–3) and the FM program (PGY 1–2), including rural and urban streams, at the University of Alberta were eligible to participate in the study. Eligible residents were contacted by their program administrator with an invitation link to participate in a survey and offered a $10 gift card for their participation. Participation was voluntary, and consent was implied as participants chose to complete the survey after reading the consenting materials.

### Survey

Before the survey was sent to participants, it was reviewed by members outside of the target population for clarity of language as part of a validity check. Participants completed the survey on Google Forms. No questions on the survey were mandatory, and participants could leave questions blank if they preferred. The items on the survey were not randomized and were conducted in 3 parts.

Part 1 of the survey covered demographic information including their year of study and residency program.

In Part 2, participants rated their confidence on a 5-point Likert scale, with 1 being not at all confident and 5 being very confident, on completing various aspects related to the neurological exam. Questions in this part were separated into the ‘non-physical’ and ‘physical’ aspects of completing an exam. For the ‘non-physical’ aspects, this included i) rating their confidence on knowing when a patient’s symptoms required a neurological exam, ii) knowing the different components of a neurological exam, iii) identifying abnormal pathology on an exam, iv) localizing, and v) forming a differential based on exam findings. In terms of the ‘physical’ aspects, participants rated their confidence on conducting the various components of the neurological exam including i) cranial nerves, ii) motor/tone, iii) reflexes, iv) sensation, and v) coordination. We defined three or less as less confident, and therefore for each question in this part of the survey, if participants rated their confidence a three or lower, they were further asked to elaborate on why they felt less confident for the particular item scored. At the end of this section, participants were asked overall what would improve their confidence on completing a neurological exam.

In Part 3, participants rated the likelihood of their colleagues (to avoid social desirability bias) skipping a clinically relevant aspect of an examination, on a 5-point Likert scale, with 1 being very unlikely to 5 being very likely, in patients with a neurological, cardiac, gastrointestinal (GI), respiratory, or musculoskeletal (MSK) complaint. For each of these specialties, participants were asked what factors may have led their peers to skipping part or all of these exams.

### Data collection and storage

All data from the survey is stored on a Google Drive and a personal device which are both password protected and only accessible by the research team. Participants with missing data were excluded from the analysis.

### Analysis

Quantitative statistical analysis was completed using IBM SPSS Statistics [18], version 26.

The quantitative data collected was evaluated with non-parametric tests. For Part 2, the ‘physical’ and ‘non-physical’ aspects of completing a neurological exam were analyzed separately. Freidman’s test was used to determine if there was a significant difference in confidence between completing the physical aspects and non-physical aspects of the exam, followed by analysis with the Wilcoxon Signed-Rank test. Pairwise comparisons were made using the Mann-Whitney U test to determine if there was a difference in confidence in Part 2 between IM and FM residents, and between different post-graduate years (PGY1 vs. PGY2 and PGY2 vs. PGY3). Freidman’s test with Wilcoxon Signed-Rank was used again in Part 3 to determine if there was a significant difference in likelihood of skipping a clinically relevant aspect of an exam between the different organ systems. We then used the Mann-Whitney U test to compare likelihood of skipping between IM and FM on neurological and MSK exams as well as comparing post-graduate years. An alpha value of 0.05 was used to determine significance.

Thematic analysis using Delve was utilized to assess the qualitative data collected from the survey. Responses were uploaded and reviewed by a single reviewer who identified a major theme when a similar response was seen 3 or more times. These themes were then fed to the Delve AI that reviewed all responses and categorized them based on themes provided and additionally identified themes not initially included. The reviewer then evaluated the output and adjusted categorizations and themes as appropriate. This was done for each free-form response question in Part 2 and 3 of the survey. As there was only a single reviewer performing the coding, inter-rater reliability could not be calculated.

### Standard protocol approvals and patient consents

This study received ethics approval through the Research Ethics Board at the University of Alberta (Pro00139344). All participants provided informed consent as outlined above.

## Results

### Part 1

There were 390 residents who were eligible to participate in the study, including 150 IM residents and 240 FM residents. A total of 97 residents participated in the study (response rate of 24.6%), with 35 from IM and 61 from FM (12 rural stream and 49 urban stream). In terms of post-graduate year, there were 36 PGY1s, 45 PGY2s, and 14 PGY3s. There were 2 respondents that did not complete the survey, and their responses were excluded from the analysis.

### Part 2

#### Non-physical aspects.

We found a difference in resident confidence between the various non-physical aspects of completing a neurological exam, χ2 (4) = 154.01, *p* < 0.001. Results can be seen in tabular format in Table 1. Overall, our analysis indicated that residents were the least confident in localizing (Median = 3; IQR 2−4) and were most confident in determining when a patient’s symptoms warranted a neurological exam (Median = 4; IQR 4−4).

**Table 1:** Resident confidence in non-physical components of the neurological examination.

Non-physical components	Median	IQR	Relative Confidence Tier	Significant Comparisons
When to examine	4	4-4	Highest	> Components (p<0.001) > Findings (p<0.001) > Differential (p<0.001) > Localizing (p<0.001)
Components	4	3-4	High–Intermediate	< When to examine (p<0.001) > Findings (p<0.001) > Differential (p<0.001) > Localizing (p<0.001)
Findings	4	3-4	Intermediate	< When to examine (p<0.001) < Components (p<0.001) > Differential (p=0.028) > Localizing (p<0.001)
Differential	4	3-4	Low–Intermediate	< When to examine (p<0.001) < Components (p<0.001) < Findings (p=0.028) > Localizing (p<0.001)
Localizing	3	2-4	Lowest	< All other domains (p<0.001 for all comparisons)

Resident confidence in conducting the non-physical aspects of the neurological exam with ranked relative confidence tiers. When to examine = knowing when a patient requires a neurological exam; Components = knowing the components of the neurological exam; Findings = identifying abnormal findings; Differential = forming a differential.

Between IM and FM residents, FM residents were more confident in forming a differential from a neurological exam compared to IM residents (*p* = 0.012; Table 2) but were otherwise comparable in the other non-physical aspects of the exam.

**Table 2:** Comparison of confidence and likelihood of skipping an examination between IM and FM residents.

	Mean Rank		*p*-value
Non-physical	IM	FM	
Symptoms	47.11	49.30	0.647
Components	47.43	49.11	0.734
Findings	42.06	52.20	0.059
Localization	34.36	50.65	0.194
Differential	**39.79**	**53.50**	**0.012**
			
Physical			
Cranial Nerves	46.31	49.75	0.504
Motor	50.66	47.26	0.517
Reflexes	49.51	47.92	0.760
Sensation	45.10	46.69	0.400
Coordination	**40.07**	**53.34**	**0.007**
			
Examination			
Neurological	49.33	48.01	0.817
Musculoskeletal	49.01	48.20	0.887

The results of the Mann-Whitney U test comparing the different aspects of the neurological examination and the likelihood of skipping a relevant aspect of an examination between the IM and FM residents. Bolded values indicate a comparison yielding a significant difference. Symptoms = knowing when a patients symptoms require a neurological examination; Components = knowing the components of the neurological examination; Findings = identifying abnormal findings; Differential = forming a differential.

When comparing the different post-graduate years, there was no significant difference in confidence among any of the non-physical aspects of the exam between PGY1 and PGY2 or PGY2 and PGY3. See Table 3.

**Table 3:** Comparison of confidence on the ‘non-physical’ aspects of the neurological examination between year of residency.

Non-Physical Component	Mean Rank		p-value
	**PGY1**	**PGY2**	
Symptoms	39.78	41.98	0.602
Components	37.08	44.13	0.111
Findings	40.82	41.14	0.945
Localization	38.21	42.28	0.407
Differential	41.53	40.58	0.844
			
	**PGY2**	**PGY3**	
When to examine	30.48	28.46	0.622
Components	29.98	30.07	0.982
Findings	30.68	27.82	0.552
Localization	31.41	25.46	0.236
Differential	31.02	26.71	0.381

The results of Mann-Whitney U Test in comparing the different years of post-graduate education on confidence in the different non-physical aspects of a neurological evaluation. Symptoms = knowing when a patients symptoms require a neurological examination; Components = knowing the components of the neurological examination; Findings = identifying abnormal findings; Differential = forming a differential.

#### Thematic analysis.

The result of the thematic analysis is presented in Table 4. The results of “knowing when to examine a patient” were not included as there were only seven responses with no major themes identified.

**Table 4:** Contributors to low confidence in the non-physical aspects of the neurological exam.

Non-physical Exam Aspects	Theme	Count	Percentage
Components	Missing/ not knowing special tests	9	43%
	Lack of practice	4	19%
	Not enough teaching	3	14%
			
Findings	Lack of exposure	24	60%
	Not confident in their own physical exam skills	8	20%
	Not enough teaching	5	13%
	Lack of practice	3	8%
			
Localizing	Lack of knowledge/ teaching	23	37%
	Lack of exposure	15	24%
	Lack of practice	15	24%
	Someone else already has/ will localize	5	8%
	Time-consuming	3	5%
			
Differential	Lack of exposure	11	29%
	Lack of knowledge	10	26%
	Lack of practice	5	13%
	Not enough teaching	3	8%
	Time-consuming	3	8%

Results of the thematic analysis examining what contributes to low confidence in the non-physical aspects of the neurological exam. The count column indicates the absolute number of responses indicating this theme, and the percentage column indicates the percent of responses received that shared this theme. Components = knowing the components of the neurological examination; Findings = identifying abnormal findings; Differential = forming a differential.

#### Physical aspects.

There was a difference in resident confidence between the various physical aspects of completing a neurological exam, χ2 (4) = 50.981, *p* < 0.001. Results can be viewed in tabular format in Table 5. Our analysis revealed that residents were most confident in conducting an examination of cranial nerves (Median = 4; IQR 4–5) and least confident with examination of reflexes (Median = 4; IQR 3–4) and sensation (Median = 4; IQR 3–4).

**Table 5:** Resident confidence across neurological examination domains.

Exam Domain	Median	IQR	Relative Confidence Tier	Significant Comparisons
Cranial Nerves	4	4-5	Highest	> Motor (p=0.003) > Reflexes (p<0.001) > Sensation (p<0.001) = Coordination (p=0.175)
Coordination	4	4-4	Intermediate–High	> Reflexes (p=0.010) > Sensation (p<0.001) = Motor (p=0.224) < Cranial Nerves (p=0.175)
Motor	4	3-4	Intermediate	> Sensation (p<0.001) > Reflexes (p<0.001) = Motor (p=0.244) < Cranial Nerves (p=0.003)
Reflexes	4	3-4	Lowest	= Sensation (p=0.083) < Cranial Nerves (p<0.001) < Motor (p<0.001) < Coordination (p=0.010)
Sensation	4	3-4	Lowest	= Reflexes (p=0.083) < Cranial Nerves (p<0.001) < Motor (p<0.001) < Coordination (p<0.001)

Resident confidence in conducting the physical aspects of the neurological examination with ranked relative confidence tiers.

When comparing IM and FM residents, FM residents were more confident testing coordination compared to IM residents (*p* = 0.007) but were otherwise comparable in the other physical aspects of the exam. See Table 2.

When comparing the post-graduate years, there was no significant difference in confidence in performing the physical aspects of the exam between PGY1 and PGY2 or PGY2 and PGY3. See Table 6.

**Table 6:** Comparison of confidence on the physical aspects of the neurological examination between year of residency.

Physical Component	Mean Rank		p-value
	**PGY1**	**PGY2**	
Cranial nerves	38.25	43.20	0.270
Motor	38.26	43.19	0.288
Reflexes	37.10	44.12	0.134
Sensation	38.97	41.69	0.576
Coordination	42.86	39.51	0.434
			
	**PGY2**	**PGY3**	
Cranial nerves	31.77	24.32	0.102
Motor	30.92	27.04	0.378
Reflexes	31.21	26.11	0.247
Sensation	29.92	30.25	0.946
Coordination	30.97	26.89	0.331

The results of Mann-Whitney U Test in comparing the different years of post-graduate education on confidence in the different physical aspects of a neurological evaluation.

#### Thematic analysis.

The result of the thematic analysis can be seen in Table 7.

**Table 7:** Contributors to low confidence in the physical aspects of the neurological exam.

Exam Component	Theme	Count	Percentage
Cranial Nerves	Lack of practice	5	45%
	Unsure of technique	3	27%
			
Motor	Lack of practice	6	25%
	Unsure of technique	7	29%
	Unsure of grading/interpretation	6	25%
	Patient factors (e.g., cooperation, consciousness)	5	21%
	Lack of anatomy knowledge	3	13%
			
Reflexes	Poor technique/ unable to elicit	13	46%
	Lack of practice	5	18%
	Lack of observed practice/ feedback	3	11%
			
Sensation	Lack of knowledge outside light touch	8	24%
	Lack of anatomy knowledge	8	24%
	Lack of practice	7	21%
	Lack of appropriate tools	5	15%
			
Coordination	Lack of practice	4	44%

Results of the thematic analysis examining what contributes to low confidence in the physical aspects of the neurological exam. The count column indicates the absolute number of responses indicating this theme, and the percentage column indicates the percent of responses received that shared this theme.

#### What would improve confidence?.

There were 63 responses to the final question in Part 2 of the survey to indicate what would make the respondents more confident conducting a neurological exam. The number of absolute responses for each theme are indicated in parentheses as well as the percentage that this theme made up of the responses. Three main themes were identified: more/improved teaching (28; 44%), more clinical exposure (27; 43%), and more practice (31; 49%). Given the number of responses, sub-themes were identified for each of the major themes above. Under teaching, respondents reported wanting more teaching done specifically by a neurologist (6; 10%) and wanted access to more educational resources (4; 6%). For clinical exposure, respondents wanted more experience seeing real neurological deficits (9; 14%). Lastly, for more practice, there was a desire for more experience with localizing (3; 5%) and having more observed hands-on practice (8; 13%).

### Part 3

There was a difference in likelihood of skipping a clinically relevant aspect of an exam between presentations from different organ systems, χ2 (4) = 183.803, *p* < 0.001. Results can be viewed in tabular format in Table 8. Our analysis revealed that residents were most likely to skip parts of neurological (Median = 3; IQR 2–3) or MSK exam (Median = 3; IQR 2–4).

**Table 8:** Likelihood of skipping clinically relevant exam component by organ system.

Clinical System	Median	IQR	Relative Skipping Risk Tier	Significant Comparisons
Neurological	3	2-3	Highest	> Cardiology (p<0.001) > GI (p<0.001) > Respirology (p<0.001) = MSK (p=0.253)
MSK	3	2-4	Highest	> Cardiology (p<0.001) > GI (p<0.001) > Respirology (p<0.001) = Neurology (p=0.253)
GI	2	1-2	Low	< Neurology (p<0.001) < MSK (p<0.001)
Respirology	2	1-2	Low	< Neurology (p<0.001) < MSK (p<0.001)
Cardiology	1	1-2	Lowest	< Neurology (p<0.001) < MSK (p<0.001)

The likelihood of skipping a clinically relevant aspect of an examination by organ system with relative risk of skipping tiers. Higher numbers indicating the exam is more likely to be skipped. MSK = musculoskeletal; GI = gastrointestinal.

When comparing IM and FM residents, there was no difference in likelihood of skipping part of a neurological (*p* = 0.817) or MSK exam (*p* = 0.887; Table 2). In comparing post-graduate years, PGY2 residents were more likely than PGY1 residents to skip a clinically relevant aspect of a neurological exam (*p* = 0.026; Table 9). PGY1 and PGY2 residents did not differ in likelihood of skipping an MSK exam. In comparing PGY2 and PGY3, there was no difference in skipping an aspect of a neurologic or MSK exam.

**Table 9:** Comparison of likelihood of skipping an examination between years of residency.

Specialty Exam	Mean Rank		p-value
	**PGY1**	**PGY2**	
Neurologic	**34.61**	**45.86**	**0.026**
Musculoskeletal	39.24	42.34	0.543
	**PGY2**	**PGY3**	
Neurologic	31.16	28.32	0.573
Musculoskeletal	30.33	31.07	0.884

The results of Mann-Whitney U Test in comparing the different years of post-graduate education on likelihood of skipping a relevant aspect of an exam. Bolded values indicate a comparison yielding a significant difference.

#### Thematic analysis.

The result of the thematic analysis can be seen in Table 10.

**Table 10:** Reasons for skipping a clinically relevant aspects of an examination.

Exam Type	Theme	Count	Percentage
Neurological	Lack of knowledge	28	41%
	Lack of confidence	22	32%
	Not enough time	23	33%
	Forgetting to do the exam	9	13%
	Someone else has/will do it	7	10%
	Reliance on imaging	6	9%
			
Cardiac	Deemed unnecessary/ irrelevant	6	23%
	Lack of knowledge	4	10%
	Lack of time	4	10%
	Forgetting to do the exam	4	10%
	Reliance on imaging/lab tests	3	8%
			
Respiratory	Reliance on imaging/lab tests	6	25%
	Lack of time	5	21%
			
GI	Patient factors (e.g., declining DRE)	12	38%
	Lack of time	5	16%
	Deemed unnecessary/ irrelevant	4	13%
	Lack of knowledge	3	9%
			
MSK	Lack of knowledge	20	40%
	Lack of confidence	15	30%
	Lack of time	7	14%
	Forgetting to do the exam	6	12%
	Reliance on imaging	6	12%
	Someone else has/will do it	4	8%

Results of the thematic analysis on reasons residents would skip a clinically relevant aspect of an examination. The count column indicates the absolute number of responses indicating this theme, and the percentage column indicates the percent of responses received that shared this theme. GI = gastrointestinal; MSK = musculoskeletal.

## Discussion

The purpose of this study was to better understand resident confidence in completing the various aspects of the neurological evaluation, understand reasons behind lack of confidence within the exam, and to assess if low confidence impacts willingness to examine neurological patients. While our study was not the first to evaluate the confidence of medical learners in the neurological exam [11,13,15], this is the first to break the exam down into its components to tease out which aspect(s) contribute to the general fear of the exam, and as well the first to evaluate skipping examination of neurological patients.

Our results demonstrated that residents had the highest confidence in determining when a patient would require evaluation from a neurological perspective, followed by knowing the components of a neurological exam. This corroborates findings from medical students that the knowledge of the essential components of the exam is likely not contributing to the ongoing anxiety surrounding a neurological evaluation [10]. As alluded to earlier, if the knowledge of the exam components is not a major contributor to anxiety of neurology, other aspects of the examination including the physical mechanics of examining a patient, or the interpretation of the examination, may be the issue. We were able to demonstrate that both IM and FM residents are generally confident in conducting the physical aspects of the neurological exam, but when it came to interpretation of exam findings (including identifying abnormal pathology, localizing, and forming a differential), our residents were less confident.

We initially hypothesized that identifying abnormal pathology would be the aspect of an evaluation that residents felt the least confident, however, we instead found that residents were least confident in localizing neurologic pathology and forming a differential. Though contrary to our initial thoughts, we felt this result was logical, as each of these steps in the neurological evaluation build on the last. This progression aligns with Bloom’s taxonomy, a framework developed to conceptualize learning outcomes [19,20]. At the bottom of the taxonomy exists knowledge and comprehension, which we feel parallels the initial part of the neurological evaluation: knowing when a patient should be examined neurologically and applying that knowledge into conducting an exam. Next is analysis, which would best align with being able to determine what is and is not abnormal on an exam. Finally, there is synthesis and evaluation, in which one would need to synthesize the findings of the exam into a cohesive localization, followed by an overall evaluation of the patient to determine a differential. If the residents begin struggling at the “analysis” stage, then it follows they will struggle with higher level concepts such as localizing and differential forming (synthesis and evaluation).

Why exactly were residents in our study less confident in interpreting the exam? From the qualitative assessment, residents told us exactly why – they do not feel knowledgeable, they do not have enough exposure to clinical neurology, and they lack practice. And, when looking at the different components of the exam, lack of exposure was by far the most prominent theme for low confidence when it came to identifying abnormal pathology, which aligned with our initial hypothesis. Interestingly, in our study, FM residents were more confident forming a differential and approached being more confident identifying abnormal pathology compared to IM residents (Table 2). We wondered if this could be related to the number of neurological problems that are encountered by FM residents versus IM residents. Neurological complaints are a common reason for patients to present to a family physician, making up to 10% of visits to primary care in the US [21]. It is important to note that in Canada, Internal Medicine is typically a consultancy service whereas in the US internists may provide primary care [22], meaning that our IM residents, compared to FM residents, would likely not have the same exposures to neurology in Canada. In addition, at our institution, it is not a requirement for IM residents to participate in a neurology rotation. This could mean that FM residents at our institution have more exposure to neurological patients, whereas the IM residents who choose not to complete a neurology rotation may see a limited number of patients with neurological issues at our tertiary site with separate neurology admitting privileges.

We also know from our study that year over year, confidence in both the physical and non-physical aspects of the exam did not improve. While this was only a comparison between cohorts and not a comparison of a cohort overtime, it raises the question if current educational practices at our institution are effective in improving resident confidence in examining neurological patients. It is also unclear how this would relate to confidence in treating or managing neurological patients. This may be an area of future study.

What are the potential consequences of our residents not being confident in the neurological exam? In the final part of our study, we had residents rate the likelihood of a peer skipping a clinically relevant aspect of an exam for the various organ systems. We chose to have participants reflect on the actions of their peers rather than themselves in hopes to avoid social desirability bias. We found that, compared to more common physical exams (cardiac, respiratory, and abdominal), residents were most likely to skip parts of a neurological or MSK exam (Table 8). A similar degree of skipping between the neurological and MSK exams was not an unexpected finding as there are many parallels between the two exams, and previous research has similarly demonstrated a lack of confidence in medical learners conducting MSK exams [23,24]. Moreover, further into post-graduate training, residents in this study became more likely to skip a relevant part of a neurological exam. When asked why these exams were skipped, residents cited low confidence as a major reason. This is alongside feeling they did not have enough time or enough knowledge. Low confidence was a particularly unique reason for skipping a neurological or MSK exam, as this was not cited as a reason for skipping exams of the other organ systems. This could indicate that low confidence in the neurological exam may prevent residents from appropriately examining patients with neurological problems, a finding that has only been suggested, but never demonstrated, in previous research [11]. This is doubly concerning when themes such as ‘someone else has/will do it’ emerged in our thematic analysis (Table 10), which may be perpetuating this idea that neurological evaluations can be skipped.

All this to say, what can we do about it? From our study, our small sample of residents have already spoken for themselves. Numerous residents in this study expressed a desire for hands-on learning, bedside teaching, and teaching direct from a neurologist. While a single rotation in neurology would provide an opportunity to address these desires, it is unlikely to improve rates of neurophobia [3]. Prior research has shown that medical learners want more longitudinal exposure to patients with neurological problems [9,25], and appear to consistently value improved clinical practice above other educational interventions [7]. This is in keeping with the findings from our study, where residents desired more exposure to clinical neurology, and appear less confident with the clinical integration of the physical exam (i.e., identifying and interpreting finding), rather than the physical mechanics of completing an exam. Taken together, one potential avenue for improving confidence could be a longitudinal neurology experience in which residents engage in bedside teaching. A recent study of IM residents has already demonstrated the feasibility in implementing longitudinal learning at the bedside [26]. Not only did the residents in this study prefer the bedside teaching over conventional lectures, they reported increased confidence when it came to approaching neurologic concerns. This study supports the idea that increased exposure to neurology will improve confidence, but it did not assess whether this longitudinal teaching format had any long-term impacts or resulted in a change in one’s behaviours. Thus, it is still unknown if increased clinical exposure to neurology would prevent skipping of neurological evaluations. This could be an area of future study.

### Limitations

There are several limitations to our study. First and foremost, this was a single centre study that focused on generalist specialties of IM and FM residents with a limited sample size. Therefore, it is unclear how our findings would generalize to other institutions, countries, or training systems, or how this would reflect the feelings of residents in other training programs. It would be interesting to replicate this study at an institution where IM or FM residents have mandatory exposure to neurology, or a study that is able to complete a subgroup analysis based on a level of exposure to neurological patients/neurology. Secondly, while this was a study to explore confidence and self-perceived likelihood of skipping examinations, there was no objective measures of examination skills or clinical behaviour, nor any correlation with competence.

We acknowledge some limitations related to our method of data collection. As with any survey methodology, our study is susceptible to response bias with various self-selection biases. Those who are passionate about neurology or those who have a strong dislike for it may have been preferentially motivated to participate. Additionally, those with strong views on the utility of the physical exam in general have been disproportionately represented. The recruitment window for our survey overlapped a transition between academic years which may have introduced heterogeneity in our study sample given differences in clinical exposures between cohorts. As we did not collect any objective measures of resident exposure to neurology, or account for differences in prior undergraduate medical training, baseline confidence levels likely varied between participants. Furthermore, in Part 3 of our survey, we asked residents to determine if they felt a clinically relevant aspect of an exam was missed. We operated under the assumption that our respondents would appreciate what would constitute clinically relevant within neurology, though we have no way of knowing what they felt was relevant. We also asked participants to rate the likelihood in which peers would skip an exam in order to overcome social desirability bias. While this approach was used to reduce bias, it could have affected accuracy of likelihood of skipping exams by our participants.

Finally, in terms of analysis, we had far less PGY3 respondents in our study. This is in part because FM residents complete a 2-year program and did make up more than half of our respondents. This may have reduced our ability to identify differences or make meaningful comparisons. Multiple comparisons were also made between the different aspects of the neurological exam, which increased our risk of Type 1 error, potentially finding significance between groups that did not exist.

## Conclusion

To summarize, our study wanted to better understand confidence of medical residents in examining neurological patients. Residents appear to be confident when it comes to determining that a patient should be examined, what that exam should look like, and physically conducting the exam, but begin to waver when the exam needs to be interpreted. Specifically, confidence was lower on identifying abnormal pathology, localizing, and forming a differential, in keeping with previous theories of learning like Bloom’s taxonomy. We did not see confidence in doing the exam improve between more senior cohorts, and confidence was low enough in performing the exam that residents were more likely to skip a necessary examination of a neurological patient. Overall, it appears that one of the largest contributors to lower confidence is poor exposure to neurology. We feel that improving resident exposure to clinical neurology could improve both resident confidence in the neurologic exam and reduce how often it is skipped in clinical medicine.
